# Investigating binding dynamics of *trans* resveratrol to HSA for an efficient displacement of aflatoxin B_1_ using spectroscopy and molecular simulation

**DOI:** 10.1038/s41598-022-06375-5

**Published:** 2022-02-14

**Authors:** Mohd Aamir Qureshi, Saleem Javed

**Affiliations:** Department of Biochemistry, Faculty of Life Sciences, Aligarh Muslim University, Aligarh, 202002 India

**Keywords:** Protein folding, Molecular biophysics, Biochemistry, Biophysics, Biotechnology

## Abstract

Resveratrol is a polyphenol belonging to the class stilbenes. The active and stable form of resveratrol is *trans*-resveratrol. This polyphenol is bestowed with numerous biological properties. Aflatoxin B_1_ is a hepato-carcinogen and mutagen that is produced by *Aspergillus* species. In this study, the interaction of *trans*-resveratrol with HSA followed by competitive dislodging of AFB_1_ from HSA by *trans-*resveratrol has been investigated using spectroscopic studies. The UV-absorption studies revealed ground state complex formation between HSA and *trans*-resveratrol. *Trans*-resveratrol binds strongly to HSA with the binding constant of ~ 10^7^ M^−1^ to a single binding site (n = 1.58), at 298.15 K. The Stern–Volmer quenching constant was calculated as 7.83 × 10^4^ M^−1^ at 298.15 K, suggesting strong fluorescence quenching ability of *trans*-resveratrol. Site markers displacement assay projected subdomain IIA as the binding site of *trans*-resveratrol to HSA. The molecular docking approach envisages the amino acid residues involved in the formation of the binding pocket. As confirmed from the site marker displacement assays, both *trans*-resveratrol and AFB_1_ binds to HSA in the same binding site, subdomain IIA. The study explores the ability of *trans*-resveratrol to displace AFB_1_ from the HSA-AFB_1_ complex, thereby affecting the toxicokinetic behavior of AFB_1_ associated with AFB_1_ exposure.

## Introduction

**Figure 1:**
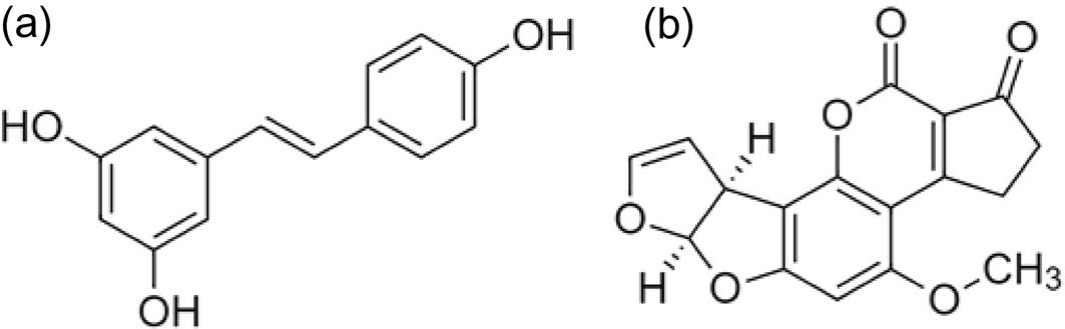
Chemical structure of **(a)** *trans*-resveratrol and **(b)** aflatoxin B_1_.

Resveratrol belongs to a class of polyphenols called stilbenes having C6-C2-C6 skeleton^1^ and is a natural stilbene present in ample amounts in grapes^2^. In nature, *Cis* and *trans* form of resveratrol are found predominantly where the *trans* form is biologically more stable and active^3^. *Trans*-resveratrol is chemically 3, 5, 4-trihydro-*trans*-stilbene, first obtained in 1939 from *Veratrum album.* It is a phytoalexin produced in response to stress or mechanical injury to a plant^4^. It is identified in approximately 70 species of plants and is predominantly found in the skin and seeds of red grapes^5^. About 50–100 μg/g concentration of *trans*-resveratrol is found in the skin of grapes. *Trans*-resveratrol is known to possess numerous properties, including anti-cancerous^5^, anti-oxidant^6^, and anti-inflammatory^7^. Its neuroprotective, antimicrobial and antifungal property has also been reported^8,9^. The effect of dietary resveratrol in AFB_1_ induced changes in broiler chicken has also been evaluated earlier^10^. The chemical structure of *trans*-resveratrol molecule is shown in Fig. 1a.

Aflatoxin B_1_ is a hazardous material and is regarded as a potent hepato-carcinogen and mutagen. It is obtained from fungus, *Aspergillus flavus*, *Aspergillus parasiticus* and *Aspergillus nomius*. However, AFB_1_ production from other species of *Emericella* has also been reported^11,12^. The crops like cereals, peanuts, and corns are exposed to these mycotoxin-producing fungi, leading to their contamination with AFB_1_^13^_._ AFB_1_ contamination is a common problem in developing countries due to the lack of proper infrastructure and facilities. It contaminates agricultural feed and poses a severe risk to the health of animals and humans. Aflatoxicosis becomes a serious concern when linked with hepatitis B (HBV) and hepatitis C (HCV) virus. Generally, ≤ 20% of the population in developing countries is affected by HBV and HCV infection. However, when HBV or HCV infection and aflatoxicosis are combined, the probability of developing the cancer increases^14^. The binding dynamics of AFB_1_ with HSA have already been documented, and it binds moderately with a binding constant of ~ 10^4^ M^−1^. The molecular structure of AFB_1_ is depicted in Fig. 1b.

HSA is a major transport protein in humans, having a molecular weight of 66.5 kDa comprising 585 amino acid residues, with single tryptophan viz Trp-214^15^. The presence of aromatic amino acid residues like Trp, Tyr, and Phe imparts HSA its fluorescent property. Nevertheless, the significant contribution is due to tryptophan residues. It is a model protein that has been explored to investigate ligand–protein interactions.

Pharmacokinetics and pharmacodynamics studies of molecules using biophysical tools are prerequisites in determining its efficacy, toxicity, and elimination process from the body. There are a number of studies exploring the hepatocellular toxicity of AFB_1_ using in vitro and in vivo approaches. Though, studies aiming at the fate of this hepato-carcinogen in the presence of serum albumin and vice versa are fewer. Recently few studies focused on the binding behavior of AFB_1_ with BSA, HSA, and chicken egg albumin^16–18^, provided information about its binding constant, site of binding, and thermodynamic parameters. Still, these studies have gaps that need to be filled by an approach that could unload the mycotoxin from the precise binding location in serum albumin, leading to its biotransformation followed by its elimination from the body. This study focuses on the very same approach using a polyphenol trans-resveratrol that could effectively dislodge the AFB_1_ from serum albumin using fluorescence spectroscopic tools. In the present study, UV-absorption spectroscopy is used to investigate the structural alterations and ground-state complex formation between HSA and *trans*-resveratrol, the fluorescence spectroscopy was used to calculate the binding constant of the polyphenol and HSA, thermodynamic parameters (ΔG, ΔH, and ΔS), followed by site marker displacement assay to examine the binding site of *trans*-resveratrol on HSA. The Circular dichroism study was performed to investigate secondary structure changes induced by *trans*-resveratrol in HSA, followed by the calculation of melting temperature (Tm). Temperature-dependent heat denaturation and unfolding profile of HSA in the presence of *trans*-resveratrol were also determined. Molecular docking analysis visualized the amino acid residues involved in the binding of *trans*-resveratrol with HSA along with major binding forces stabilizing the interacting entities. In silico amino acid substitution study was performed to ascertain the role of Trp-214 in the binding process of the ligand to the protein molecule. After confirming the binding location of the polyphenol, a comparative analysis of the fluorescence quenching strength of *trans*-resveratrol versus AFB_1_ was established for HSA. Later on, the displacement assays were performed to analyze the dislodging potential of *trans*-resveratrol to displace AFB_1_ from the HSA and vice versa. The outcome of this study will help the researchers to understand the kinetics and dynamics of the binding process of *trans*-resveratrol and AFB_1_.

## Results and discussion

### Spectroscopic studies

**Figure 2:**
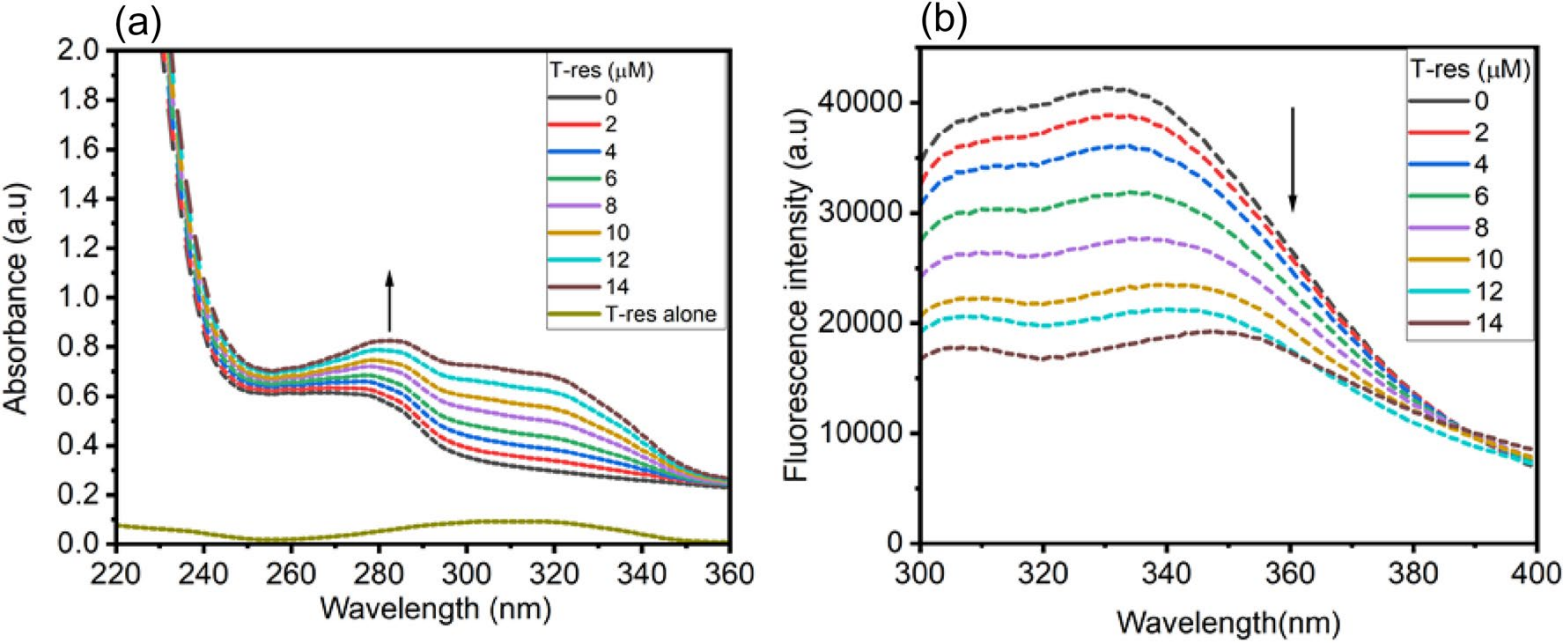
**(a)** UV-absorption spectrum of HSA (5 µM), and **(b)** fluorescence emission spectrum of HSA (5 µM) in the presence of an increasing concentration of *trans*-resveratrol (0–14 µM) at 298.15 K and pH 7.4. λ_ex_ of HSA = 280 nm. The concentration of *trans*-resveratrol alone is 2 µM.

The binding and interactive mode of flavonoid *trans*-resveratrol with HSA was investigated before its use as a dislodging agent for HSA bound AFB_1_. The binding behavior was studied using spectroscopic tools like fluorescence spectroscopy and UV–visible spectroscopy. UV-absorption spectroscopy was used to ascertain the structural changes induced by *trans-*resveratrol upon binding with HSA. The UV-absorption property of HSA is by virtue of its aromatic amino acids (Trp, Tyr, and Phe) that impart a strong UV absorption signal at 280 nm^19^. Nevertheless, *trans*-resveratrol shows the absorption signal at 319 nm, as shown in Fig. 2a. From the Fig. 2a, it is clear that in the presence of an increasing concentration of *trans*-resveratrol, the hyperchromic effect was observed at absorption maxima (λ_max_) of HSA, coupled with bathochromic shift, suggesting structural alterations in the native structure of HSA and a ground state complex formation between *trans*-resveratrol and HSA^20^. The absorption spectrum also gives a clue about the existence of static quenching between *trans*-resveratrol and HSA, since in static type of quenching, the absorption spectra of native protein changes in the presence of a ligand molecule, however in dynamic quenching, it remains unaffected^21,22^. Fluorescence spectroscopy was performed to get insight into the binding and thermodynamics parameters associated with the interaction of *trans*-resveratrol with HSA. When excited at 280 nm, a strong fluorescence quenching was observed in the fluorescence emission spectrum of HSA in the presence of an increasing concentration of *trans*-resveratrol (0–14 µM) as shown in Fig. 2b. HSA consists of fluorophores, critical for the fluorescent property of the protein viz, Trp, Tyr, and Phe, where the major contribution is from Trp-214 residue^23^. Quenching is accompanied by redshift suggesting structural and conformational alterations in the native structure of HSA in the presence of *trans*-resveratrol. Bathochromic shift is the result of the increase in the polarity around fluorophores in HSA in the presence of *trans*-resveratrol^24^.

**Figure 3:**
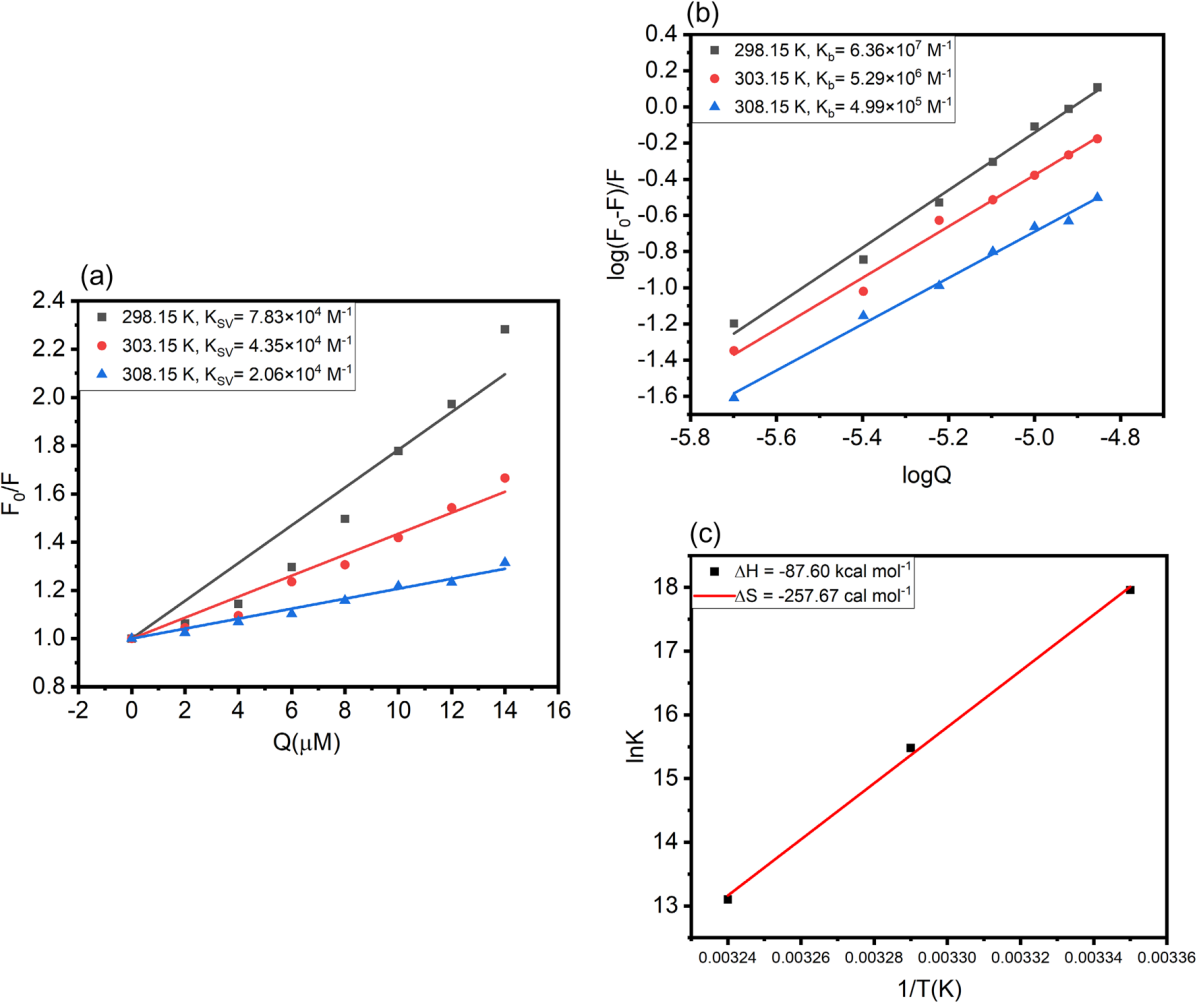
**(a)** Plot of F_0_/F versus Q for the calculation of Stern–Volmer quenching constant (K_SV_), **(b)** plot of log (F_0_/F)/F versus log (Q) for the calculation of binding constant (K_b_), **(c)** Van’t Hoff plot of lnK versus 1/T (K) for the calculation of thermodynamic parameters for HSA-T-res system, at 298.15, 303.15 and 308.15 K and pH 7.4.

Stern–Volmer quenching constant (K_SV_) for binding *trans*-resveratrol with HSA was calculated according to Eq. (1) ^25^. There is linearity between the concentration of T-res (Q) and F_0_/F, as shown in Fig. 3a, at 298.15, 303.15, and 308.15 K. With the rise in temperature, a decrease in the slope of the Stern–Volmer plot is observed, the binding between HSA and *trans*-resveratrol also destabilizes with the increase in the temperature, suggesting a static mode of fluorescence quenching and quashing the existence of dynamic quenching mechanism operating between HSA and *trans*-resveratrol^26^. Nevertheless, from Fig. 3a, if we look at the F_0_/F vs Q plot at 298.15 K, at a higher concentration of *trans*-resveratrol, the F_0_/F vs Q plot is shifted towards y axis, envisaging a mixed type of fluorescence quenching mechanism at 298.15 K, however, the plot shows the complete straight line at 303.15 and 308.15 K. The major contribution is for static nature of fluorescence quenching since the K_SV_ value decreases with increase in the temperature Further confirmation of the static quenching mechanism was done by calculating the bimolecular quenching constant (K_q_) using Eq. (3)^25^ of the “Methods” section. The numerical value of K_q_ obtained at three different temperatures, as shown in Table 1, is greater than the maximum scattering collision constant whose value is 2 × 10^10^ M^−1^ s^−1^, thus confirming the existence of static quenching and ground-state complex formation between HSA and *trans*-resveratrol^24,27^. In a protein and ligand interaction, three different natures of fluorescence quenching can be observed, static, dynamic, and mixed (both static and dynamic) quenching. These different kinds of quenching patterns can be differentiated based on the K_SV_ values obtained at different temperatures. Static quenching is marked by ground-state complex formation between ligand molecule and protein^28^, followed by decreases in the K_SV_ values with an increase in the temperature. The decrease in the K_SV_ is the result of a decrease in the ligand and protein stability with the rise in the temperature. However, in the dynamic nature of fluorescence quenching, the K_SV_ value increases with the rise in the temperature^29,30^. Dynamic quenching results from the collision of the fluorophore and the ligand molecule. The mixed nature of fluorescence quenching displays both the properties of static and dynamic quenching^31^.

**Table 1:** Tabular representation of Stern–Volmer quenching constant (K_SV_), bimolecular quenching constant (K_q_) for the HSA-T-res system at 298.15, 303.15, and 308.15 K and pH 7.4.

Temperature (K)	K_SV_ (M^−1^)	K_q_ (M^−1^ s^−1^)	R^2^	SD
298.15	7.83** × **10^4^	7.83** × **10^13^	0.993	0.005
303.15	4.35** × **10^4^	4.35** × **10^13^	0.998	0.001
308.15	2.06** × **10^4^	2.06** × **10^13^	0.999	0.007

The binding constant (K_b_) was calculated at three different temperatures, viz 298.15, 303.15, and 308.15 K using Eq. (4)^25,32^, mentioned in the “Methods” section. The plot of log (F_0_ − F)/F *versus* log (Q) is shown in Fig. 3b. The plot is a straight line, and the value of K_b_ was calculated to be 6.36 ± 0.32** × **10^7^ M^−1^ at 298.15 K. Such a high binding constant is an indication of strong binding affinity between HSA and *trans*-resveratrol. The values of K_b_ and number of binding sites (n) at three different temperatures are reported in Table 2.

**Table 2:** Tabular representation of binding constant (K_b_) and thermodynamic parameters like ΔH, ΔG, and ΔS for HSA-T-res system at three different temperatures, 298.15, 303.15, and 308.15 K.

Temp (K)	K_b_ (M^−1^)	R^2^	n	ΔG (kcal mol^−1^)	ΔH(kcal mol^−1^)	ΔS(cal mol^−1^ K^−1^)
298.15	6.36 ± 0.32** × **10^7^	0.990	1.58 ± 0.006	−10.63	−87.60	−257.67
303.15	5.29 ± 0.33** × **10^6^	0.987	1.42 ± 0.006	−9.32		
308.15	4.99 ± 0.23** × **10^5^	0.992	1.27 ± 0.004	−8.03		

The binding affinity of AFB_1_ towards HSA has already been reported earlier by Tan et al.^33^. AFB_1_ binds to HSA in subdomain IIA with a binding constant ~ 10^4^ M^−1^. The binding constant (K_b_) for AFB_1_- HSA system is lower than the HSA-T-res system.

The Gibbs free energy (ΔG), enthalpy change (ΔH), and entropy change (ΔS) are the essential thermodynamic parameters that portray the spontaneity and favorability of a chemical reaction. These thermodynamic values are calculated using Eqs. (6) and (7) of the “Methods” section. Figure 3c shows the lnK versus 1/T (K) plot, the intercept and slope of the plot were used in the calculation of ΔS and ΔH, which was calculated to be −257.67 cal mol^−1^ K^–1^ and −87.60 kcal mol^−1^, respectively. The magnitude of both ΔS and ΔH is negative, signifying hydrogen bonding and van der Waals interaction as the major forces acting in HSA and *trans*-resveratrol complex stabilization. The principal forces acting between the protein and ligand molecule are hydrogen bonding and van der Waals interaction when ΔS < 0 > ΔH^34^. We also calculated the value of ΔG for the HSA-T-res system that was found to be negative, suggesting a favorable and spontaneous process of *trans*-resveratrol binding with HSA. All the thermodynamic parameters are reported in Table 2.

### Location of the binding site of *trans*-resveratrol in HSA using site markers displacement assay

**Figure 4:**
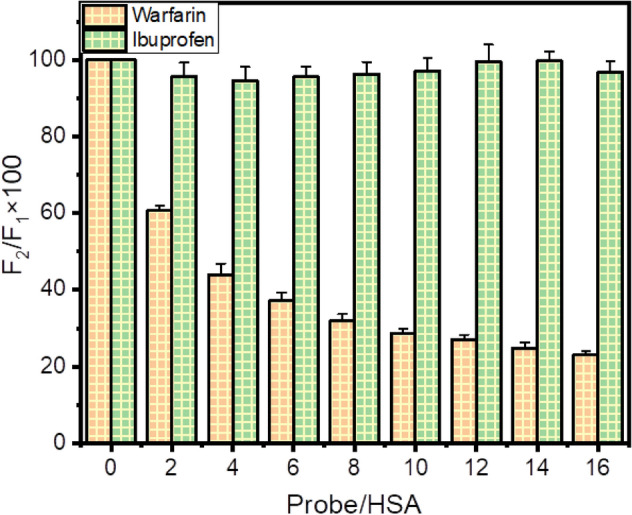
Graphical representation of site marker displacement assay for the location of binding site of *trans*-resveratrol on HSA at 298.15 K and pH7.4, warfarin, and ibuprofen site markers were used for subdomain IIA and subdomain IIIA specific binding, respectively. At the level of 0.05 (p < 0.05), the data is significant. Error bar represents the standard deviation value (mean ± SD).

Our molecule of interest, *trans*-resveratrol, binds to HSA with a much higher affinity as compared to AFB_1_, hence possessing the potential to compete with AFB_1_. We further explored the binding site of *trans-*resveratrol on HSA using site markers, warfarin, and ibuprofen. These two site markers are routinely used probe molecules to locate the binding site of a small molecule on protein^35^. Most of the ligand binds to the protein at Sudlow’s site I (subdomain IIA) and Sudlow’s site 2 (subdomain IIIA). Warfarin binds to subdomain IIA, and ibuprofen binds to subdomain IIIA. The binding location of AFB_1_ on HSA has already been studied by Tan et al. and Poor et al.^17,33^, confirming Sudlow’s site 1 as the binding pocket of AFB_1_ on HSA. If *trans-*resveratrol and AFB_1_ share the same binding site on the protein molecule, *trans*-resveratrol, by virtue of its higher binding constant (K_b_) for HSA than AFB_1,_ could easily displace the mycotoxin from the HSA and increase its availability in the body in free form rather than bound form. The percentage displacement was calculated from the plot of F_2_/F_1_ × 100 versus probe/HSA, obtained using Eq. (7) of the “Methods” section^36,37^. From Fig. 4, it is inferred that the percentage displacement of *trans*-resveratrol by warfarin is more prominent than ibuprofen, which provides a clue that *trans*-resveratrol binds at the site in HSA where warfarin binds, suggesting subdomain IIA or Sudlow’s site 1 as the binding site of *trans*-resveratrol in HSA. Various studies on ligand and protein interactions have used site markers displacement assays as a reliable method for locating the binding site of the ligand of interest in protein molecules^38,39^.

### Circular dichroism measurement

**Figure 5:**
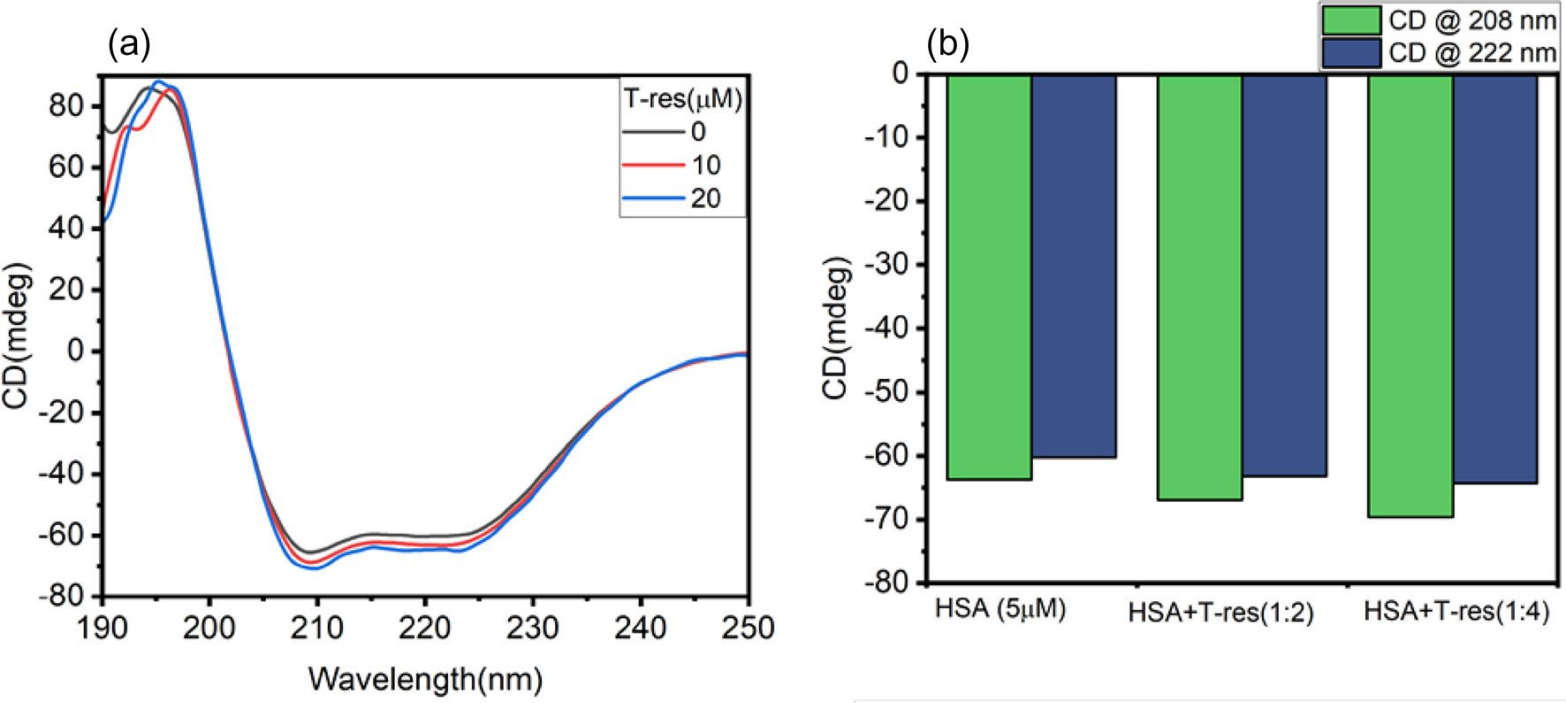
**(a)** Far UV-CD spectra of HSA (5 µM) in the presence of a different concentration of *trans*-resveratrol with HSA (10 µM and 20 µM) at 298.15 K and pH 7.4. **(b)** Bar graph showing the CD values in mdeg, at 280 and 222 nm, for HSA alone and HSA in the presence of different molar ratio of *trans-*resveratrol (1:2 and 1:4).

Circular dichroism is a valuable tool for deciphering the conformational or secondary structure change in the protein induced by a ligand^40^. It is a routinely used technique involved in ligand and protein interaction to investigate the nature of the binding between protein and molecules^41^. Interaction of chromophores in the protein molecule, in an asymmetric milieu, with the polarized light results in CD signals^42^. Peptide bonds absorb polarized light in the far UV–region^43^. The far UV-CD signal of HSA with predominant alpha helix exhibits two negative ellipticity at 208 and 222 nm as a consequence of n → π* and π → π* transition^44^. From Fig. 5a, it is observed that the native HSA exhibited two peaks at 208 and 222 nm, suggesting the predominance of the alpha helix. The MRE value at 208 nm and percentage alpha helix was calculated using Eqs. (8) and (9) of the “Methods” section^38,45^.

Native HSA showed 61.39% alpha-helical content. In the presence of *trans*-resveratrol (10 and 20 µM), the alpha-helical content was changed to 65.07 and 68.24%, respectively. Figure 5a,b indicate *trans*-resveratrol induced conformational changes and increase in alpha-helix in HSA, suggesting stabilization of native structure of HSA in the presence of *trans*-resveratrol. Lower concentrations of *trans*-resveratrol (2–8 µM) induced insignificant changes in the secondary structure of HSA. Figure 5b shows the CD values at 208 and 222 nm for HSA in the absence and presence of *trans*-resveratrol, which depicts a clear picture of the increase in CD (mdeg) values corresponding to increase in an alpha helix at 10 and 20 µM *trans*-resveratrol. Some phytochemicals and drug molecules, on interaction with albumins, have shown to increase the alpha helix and thereby its stability, and it is due to an increase in the extent of hydrogen bonding in the protein molecules^46–48^.

**Figure 6:**
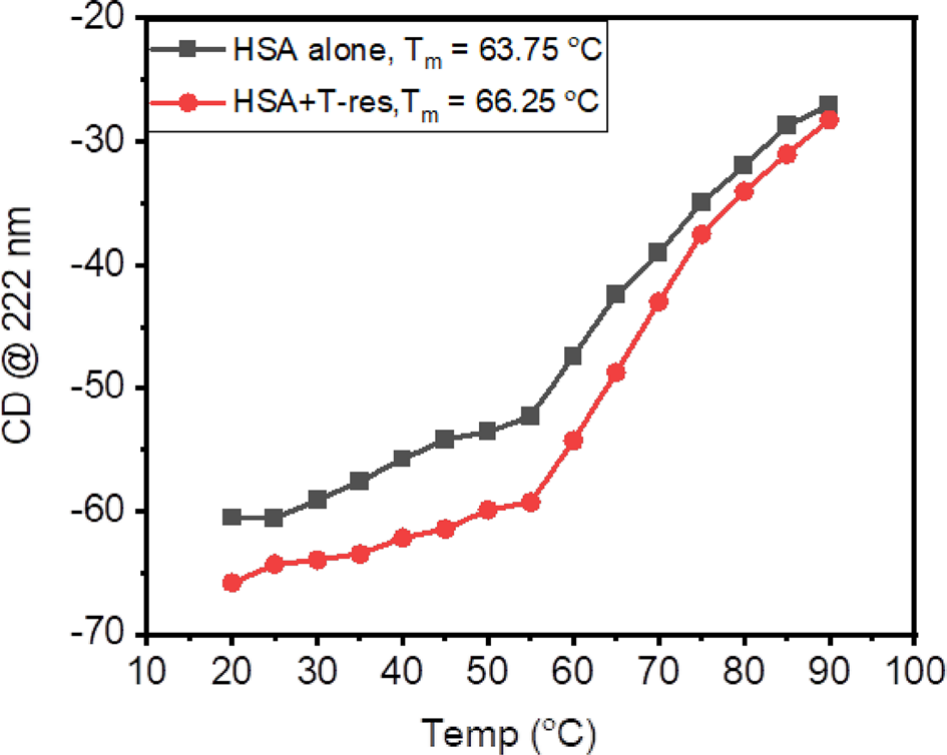
Thermal melting profile of HSA (5 µM), in the presence of *trans*-resveratrol (20 µM) for the calculation of T_m_ at the temperature range of 20 °C to 90 °C and pH 7.4.

Thermal stability of HSA in the presence of *trans*-resveratrol was also investigated using CD spectroscopy, by measuring changes in CD signal at 222 nm by sigmoidal fitting, as a function of temperature (20–90 °C). Hydrophobic interactions are the major contributors to the folding mechanism of protein. However, other factors like hydrogen bonding and electrostatic interactions also play significant roles in stabilizing protein structure^49^. T_m_ is the midpoint transition temperature at which the equilibrium is maintained between folded and unfolded form^50^. The thermal stability of the protein is directly proportional to its T_m_ value. In other words higher the T_m_ value more is the thermal stability of the protein^51^. The native HSA exhibited T_m_ values of 63.75 °C. However, in the presence of *trans*-resveratrol, it was increased to 66.25 °C. The increase in the T_m_ value from 63.75 °C to 66.25 °C confirms the *trans*-resveratrol-assisted folding of HSA. The thermal unfolding experiment further explores that the thermal stability of HSA is increased in the presence of *trans*-resveratrol. Certain drugs which bind to subdomain IIA, like warfarin and virstatin have been known to increase the T_m_ of HSA, as reported in earlier studies^52,53^. Figure 6 shows the melting profile of HSA in the presence of *trans*-resveratrol.

**Figure 7:**
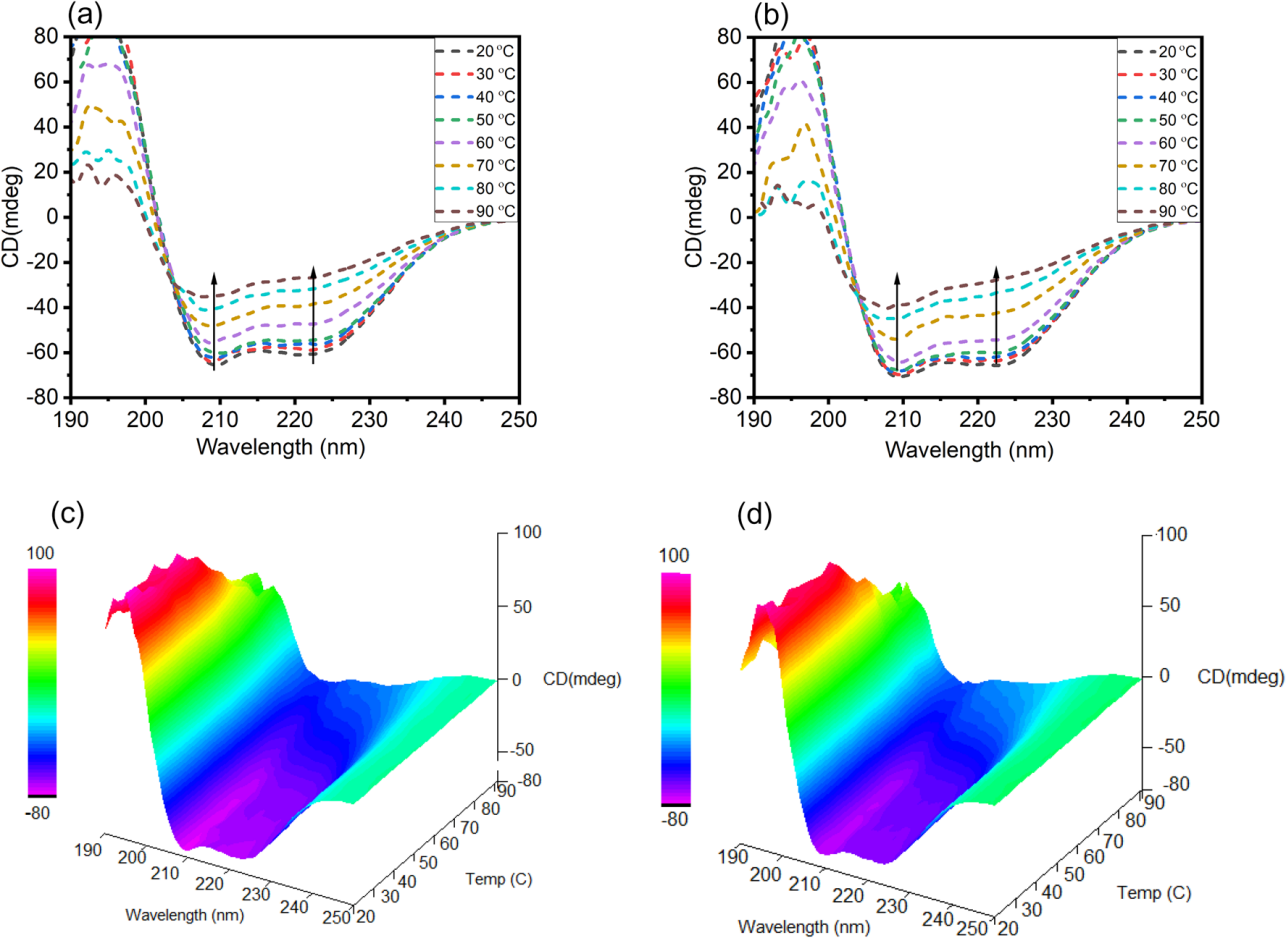
Far-UV CD spectra of **(a)** HSA alone, **(b)** HSA in the presence of *trans*-resveratrol (20 µM) as a function of temperature ranging from 20 to 90 °C. Three dimensional far-UV CD spectra of **(c)** HSA alone and **(d)** HSA in the presence of *trans*-resveratrol (20 µM) as a function of temperature ranging from 20 to 90 °C.

Far UV- three dimensional CD spectra of HSA in the absence and presence of *trans*-resveratrol as a function of temperature were also plotted to envisage the ligand-induced structural and conformational perturbation of HSA at each temperature ranging from 20 to 90 °C. From Fig. 7, it is clear that with each rise in temperature from 20 to 90 °C, the negative ellipticity at 208 and 222 nm decreases, suggesting the unfolding of HSA as a function of temperature. In the presence of 20 µM *trans*-resveratrol, the alpha-helical content of HSA is protected at each rise in temperature, suggesting the *trans*-resveratrol mediated stabilization of the secondary structure of native HSA. From Table 3, it is evident that at 20 °C, native HSA showed 61.30% alpha-helix, and at 90 °C, it was reduced to 27.69% as a result of unfolding and temperature-induced denaturation. However, *trans*-resveratrol bound HSA at 20 °C exhibited 67.58%, higher than the HSA alone, at each increment in the temperature; HSA bound *trans*-resveratrol showed higher alpha-helix as compared to HSA alone at the same temperature. The drastic decrease in the alpha helix in HSA as a function of temperature is attributed to the reduction in the hydrogen bonding in the amino acids. Nevertheless, *trans*-resveratrol proved to be effective in protecting the unfolding of HSA, thereby restoring hydrogen bonding in the amino acid residues at a given temperature range. Figure 7 reflects the far UV-CD spectra of HSA in the presence of *trans*-resveratrol from 20 to 90 °C.

**Table 3:** Table showing alpha-helical content in HSA and HSA + T-res system as a function of temperature ranging from 20 to 90 °C at pH 7.4.

Temp(^o^C)	HSA (5 µM)	HSA + T-res (20 µM)
20	61.30%	67.58%
30	60.72%	66.54%
40	58.85%	66.98%
50	55.60%	65.34%
60	51.32%	59.21%
70	42.73%	49.47%
80	34.84%	38.93%
90	27.69%	34.32%

### Displacement of AFB_1_ from HSA by *trans*-resveratrol

**Figure 8:**
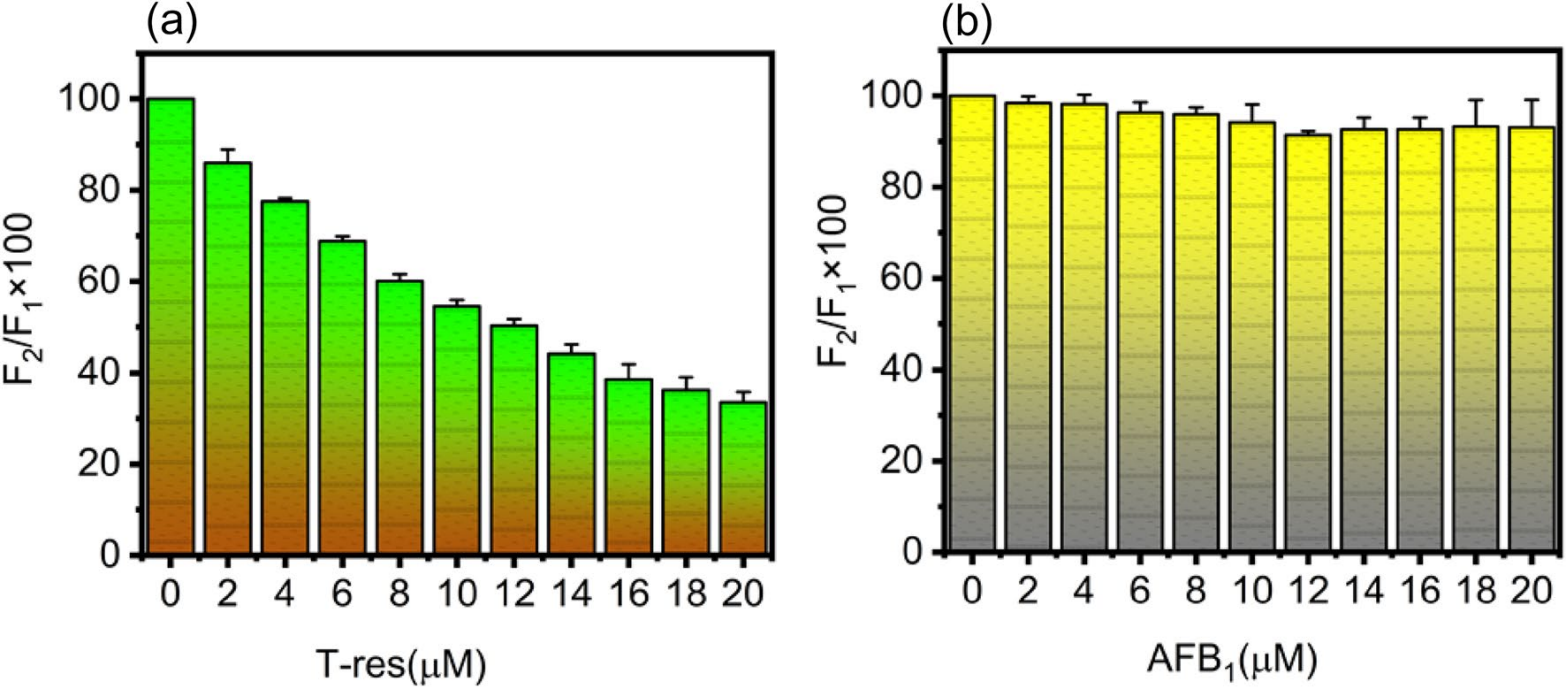
Graphical representation of **(a)** AFB_1_ displacement by *trans*-resveratrol in HSA-AFB_1_ system, **(b)** *trans*-resveratrol displacement by AFB_1_ in HSA-T-res system. At the level of 0.05 (p < 0.05), the data is significant. Error bar represents the standard deviation value (mean ± SD).

After confirming that AFB_1_ and *trans-*resveratrol shares the same binding site, subdomain IIA in HSA, displacement assay was followed to check the dislodging potential of *trans*-resveratrol against AFB_1_ competing for the same binding pocket. In Fig. 8a, it is shown that each increasing concentration of *trans*-resveratrol (0–20 µM), displaces the AFB_1_ bound to HSA from the HSA-AFB_1_ system. However, when the displacing potential of AFB_1_ against *trans*-resveratrol was studied for the HSA-Tres system, as shown in Fig. 8b, AFB_1_ failed to dislodge *trans*-resveratrol from HSA, indicating the inability of AFB_1_ to compete for albumin sharing the same binding site with *trans*-resveratrol. From Fig. 8b, it is clear that the increasing concentration of AFB_1_ (0–20 µM) has no effect on the percentage displacement of *trans*-resveratrol bound to HSA.

### Comparative analysis of the binding potentials of *trans*-resveratrol and AFB_1_ to HSA

**Figure 9:**
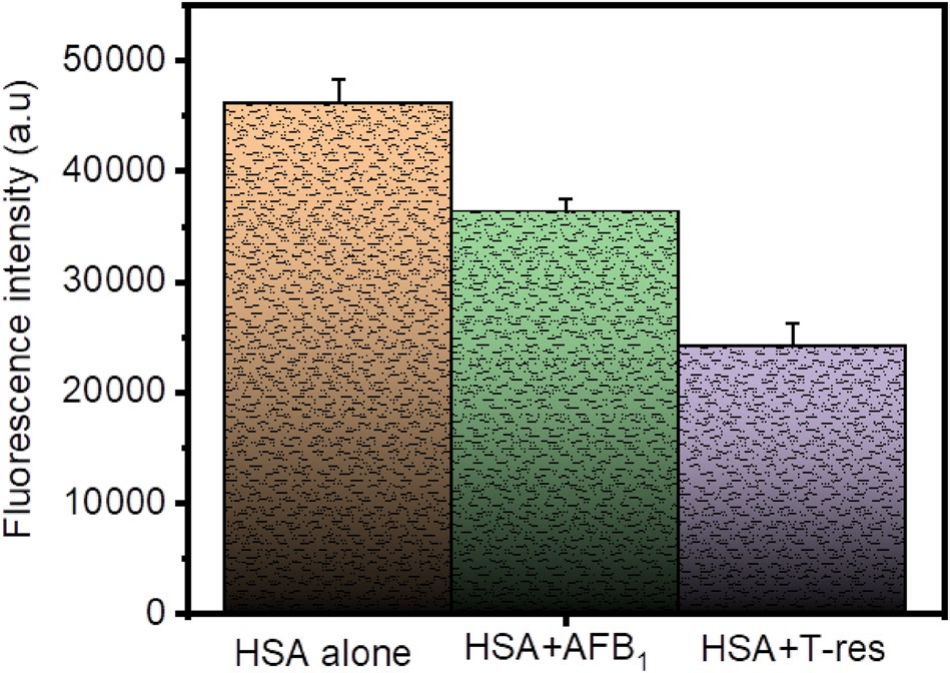
Graphical representation of the fluorescence intensity of HSA in the presence of *trans*-resveratrol and AFB_1_, excited at 280 nm. The concentration of HSA is 5 µM and the concentration of T-res = AFB_1_ = 14 µM. At the level of 0.05 (p < 0.05), the data is significant. Error bar represents the standard deviation value (mean ± SD).

We also performed the comparative analysis of the effect of AFB_1_ and *trans*-resveratrol on HSA, as depicted in Fig. 9. From the figure, based on the values of the fluorescence intensities, the emission spectrum of HSA underwent more quenching in the presence of *trans*-resveratrol than AFB_1_. The decrease in the fluorescence intensity is more in HSA + T-res as compared to HSA + AFB_1_. To further gain insights into the dislodging potential of *trans*-resveratrol, competing for HSA, equal concentrations of AFB_1_ and *trans*-resveratrol were used for displacing *trans*-resveratrol bound HSA and AFB_1_ bound HSA, respectively. From Table 4, it is evident that HSA alone (5 µM) showed fluorescence intensity of 47,776.6, the fluorescence intensity in the presence of AFB_1_ and *trans*-resveratrol was decreased to 37,322.6 and 24,784.8, respectively. The fluorescence intensity of HSA for the HSA + T-res + AFB_1_ system (Table 4) insignificantly differed from the fluorescence intensity of the HSA + T-res system, thereby confirming the inability of AFB_1_ to displace *trans*-resveratrol from HSA-T-res. Nevertheless, the fluorescence emission intensity of HSA for the HSA + AFB_1_ + T-res system (Table 4) significantly changed from the fluorescence emission intensity of the HSA + AFB_1_ system, elucidating the potentiality of *trans-*resveratrol to dislodge the AFB_1_ bound to albumin and apprehending its binding site.

**Table 4:** The effect of AFB_1_ and *trans-*resveratrol on the fluorescence intensity of HSA at λex = 280 nm, T = 298.15 K, and pH = 7.4.

System	Fluorescence intensity
HSA alone	47,776.6 ± 183.17
HSA + AFB_1_	37,322.6 ± 236.79
HSA + AFB_1_ + T-res	17,301.3 ± 250.46
HSA + T-res	24,784.8 ± 153.83
HSA + T-res + AFB_1_	22,610.6 ± 356.61

The concentration of HSA alone was taken as 5 µM and the concentration of T-res = AFB_1_ = 14 µM.

### Molecular docking and amino acid substitution studies

**Figure 10:**
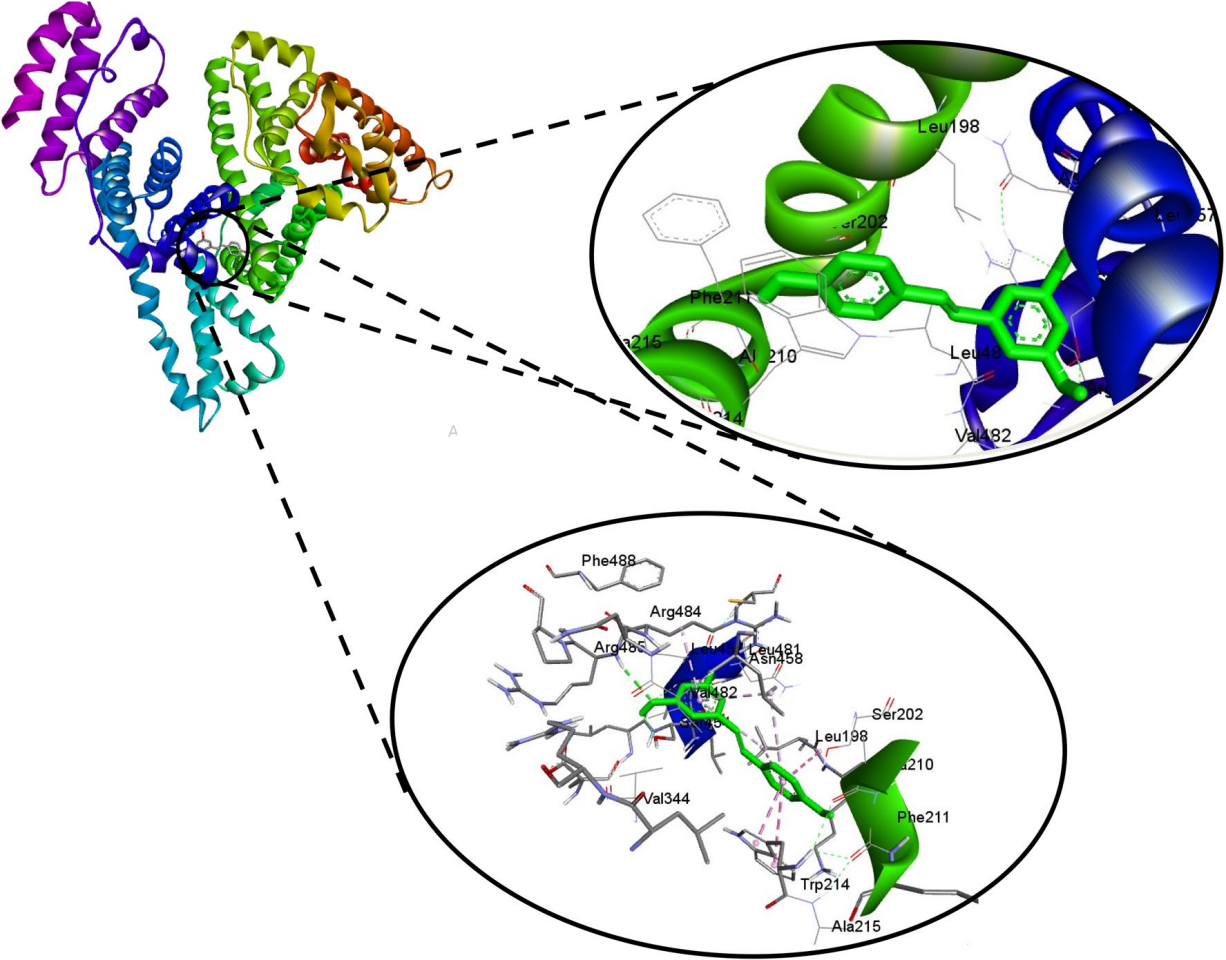
Molecular docking of *trans*-resveratrol with HSA showing the best docked pose, site of binding and the amino acid residues involved in the complex stabilization.

**Figure 11:**
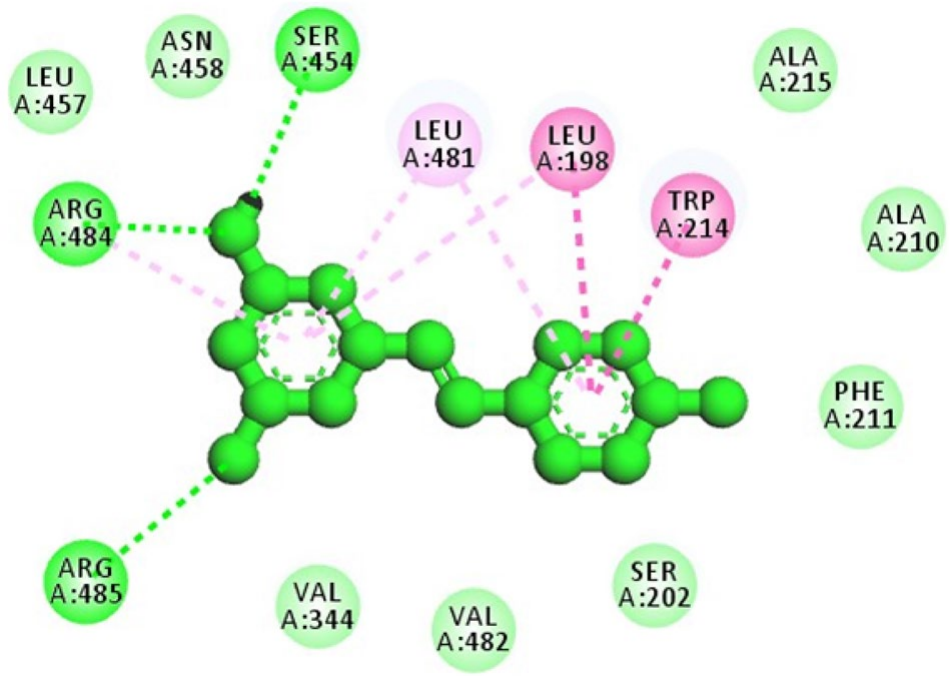
Two dimensional representation of the amino acid residues binding *to trans-*resveratrol molecule.

Molecular docking is a powerful computational approach to investigate ligand binding to the protein molecule at the atomic level^54^. These in silico tools were used to explore and recognize the binding site of *trans*-resveratrol in HSA, corroborate the findings of spectroscopic studies, and get insight into the amino acid residues involved in the binding of *trans*-resveratrol with HSA. Figure 10 shows the best-docked pose and Sudlow’s site 1 as the binding pocket for the binding of *trans*-resveratrol with HSA. The binding energy for the interaction as calculated from the docking is −7.76 kcal mol^−1^. If we look at Fig. 10, Trp-214 is present in the binding pocket, and it has a role in the stabilization of HSA *trans-*resveratrol complex. The amino acid residues surrounding the *trans*-resveratrol molecule are shown in Table 5. Figure 11 depicts the 2D picture of the amino acid residues in the vicinity of the *trans*-resveratrol and the nature of bonds formed between them. The hydrogen bonding and van der Waals interactions are best illustrated by the 2D pot of the HSA and *trans*-resveratrol interaction. Previous studies on the interaction of AFB_1_ with HSA also confirmed Sudlow’s site 1 as the binding pocket for AFB_1_ using in silico approach^17^, and the amino acids residues involved in the binding are in very close proximity to that involved in HSA and *trans*-resveratrol binding in the same Sudlow’s site 1. The van der Waals + hydrogen bonding + desolvation energy for HSA and *trans*-resveratrol complex was −9.07 kcal/mol, much higher than their electrostatic energy of −0.19 kcal/mol, thus proposing hydrogen bonding and van der Waals interaction as the major forces stabilizing the HSA and *trans*-resveratrol complex. This further corroborates our thermodynamic findings. Since the binding affinity of *trans-*resveratrol is much higher than the AFB_1_*, trans*-resveratrol is able to displace bound AFB_1_ to HSA for competing the binding site. Similar binding sites (subdomain IIA) have also been reported in a study of the binding of oxyresveratrol to HSA^39^.

**Table 5:** Docking parameters like binding energy and amino acids involved in T-res-HSA and T-res- HSA mutant interaction.

Protein–ligand system	Amino acid residues involved in stabilizing HSA-T-res complex	Binding energy of the complex
(a) T-res-HSA	Ala-215, Ala-210, Phe-211, Ser-202, Val-482, Val-344, Trp-214, Leu-198, Leu-481, Ser-454, Asn-458, Leu-457, Arg-484 and Arg-485	−7.76 kcal mol^−1^
(b)T-res-HSA (Trp-214 substituted with Gly)	Arg-484, Ser-454, Leu-198, Leu481, Lys-199, Ser-202, Ala-215, Ala-210, Gly-214, Phe-211, Asn-458, Leu-457, Val-344, Val-482, Leu-347	−7.06 kcal mol^−1^
(c) T-res-HSA (Trp-214 substituted with Val)	Lys-190, Asn-429, Ala-194, Asp-108, Tyr-148, Ala-194, His-146	−6.29 kcal mol^−1^

**Figure 12:**
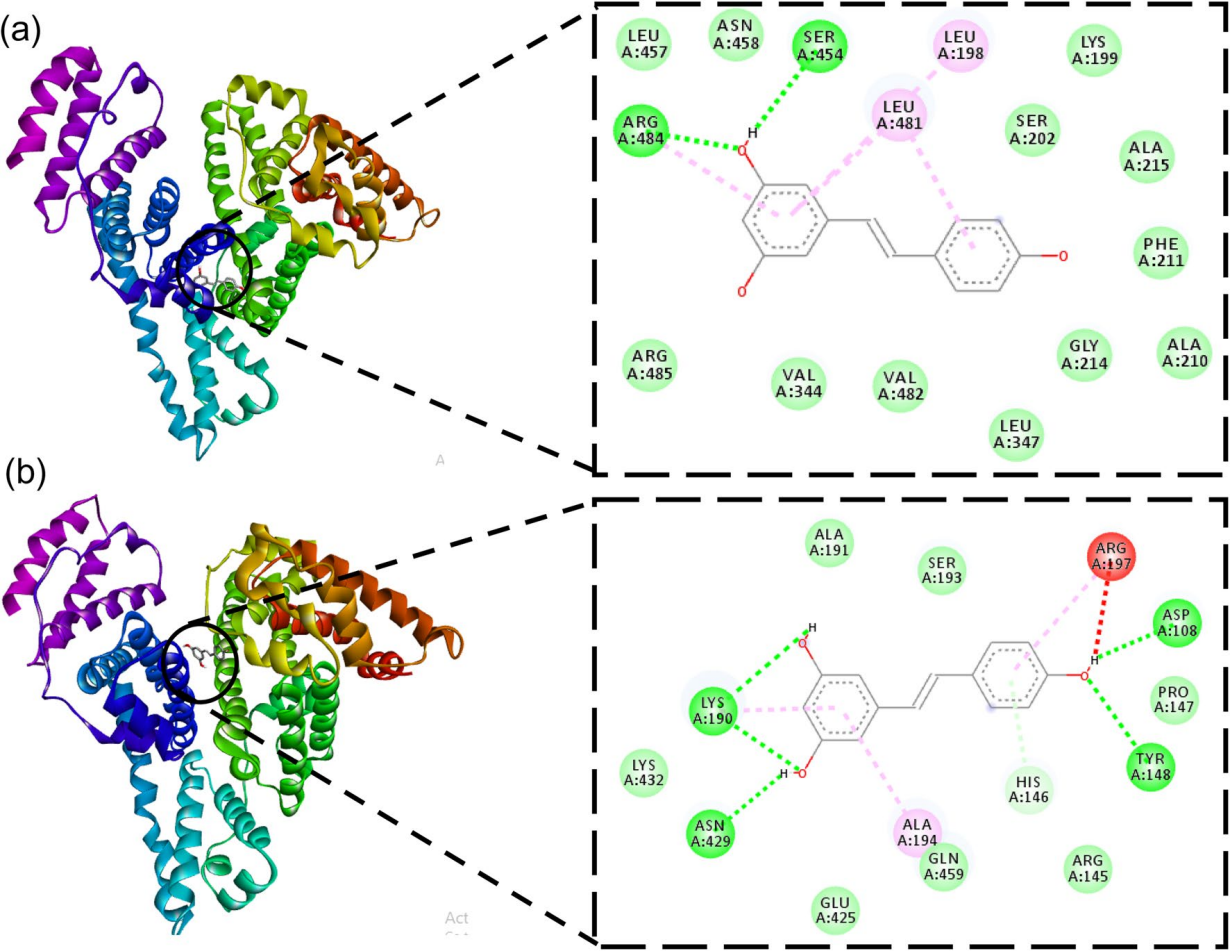
Molecular docking analysis for the binding of Trp-214 mutant HSA to *trans*-resveratrol. **(a)** Trp-214 is substituted with glycine residue. **(b)** Trp-214 is substituted with valine residue.

We further explored the binding of mutated HSA with *trans*-resveratrol. In mutated HSA, Trp-214 was substituted with neutral amino acid glycine to elucidate the changes in the binding fashion of *trans*-resveratrol with mutant HSA as compared to non-mutated HSA. Glycine was substituted in place of Trp-214 because glycine, the simplest amino acid, is also neutral. The molecular docking study revealed that the complex of *trans*-resveratrol with mutant HSA was less stabilized than the non-mutated complex because the binding energy of the mutated HSA complex was less negative compared to the non-mutated complex. The binding energy of the complex and the amino acid residues involved in the stabilization of the mutated HSA-*trans*-resveratrol complex is shown in Table 5. Interestingly, the binding site of *trans-*resveratrol was located at the same subdomain IIA as it was with non-mutated HSA. However, the number of hydrogen bonds was reduced to 2 only, as shown in Fig. 12. Gly-214 was not involved in any kind of bonding with *trans*-resveratrol. A mutation study was also performed by substituting the hydrophobic amino acid valine in Trp-214. Surprisingly, the binding site of *trans*-resveratrol on HSA was different as compared to native non-mutated HSA, and HSA with Trp-214 substituted with Gly. The binding energy of the complex was also less negative. The study with mutated HSA was significant since it investigates the role of Trp-214 during the stabilized complex formation between HSA and *trans-*resveratrol. The substitution of Trp-214 with either Gly or Val did not result in the highly stabilized complex formation as compared to the non-mutated HSA. The binding of the mutated HSA complex with *trans*-resveratrol was less negative as compared to the native HSA. The amino acid residues that form the binding pocket for mutant HSA and *trans*-resveratrol interaction are shown in Table 5. Figure 12 shows the best-docked pose of binding of *trans*-resveratrol with mutated HSA. Table 5 represents the amino acid residues involved and binding energies in the formation of mutated HSA-*trans*-resveratrol complex.

### Molecular dynamic simulation studies

**Figure 13:**
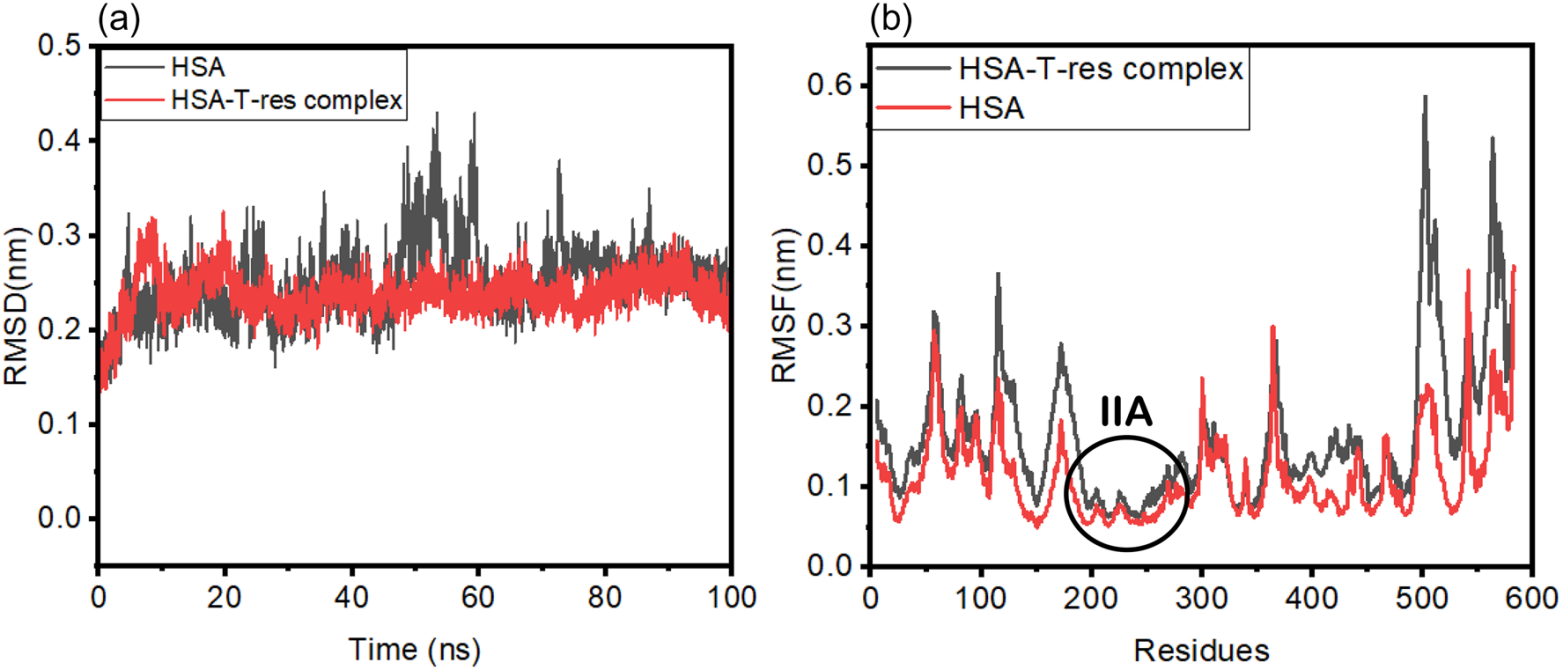
MD simulation analysis of HSA and *trans-*resveratrol binding interaction showing **(a)** RMSD plot for 100 ns simulation, **(b)** RMSF plot showing the fluctuations of the residues in HSA and HSA-T-res complex.

Molecular dynamic (MD) simulation studies were carried out to get insights into the dynamic nature of *trans*-resveratrol binding to HSA over the simulated period up to 100 ns. For the investigation of the system stability, root mean square deviations (RMSD) values of HSA backbone (C–Cα–N) and HSA-T-res complex were calculated, and the graph was plotted from 0 to 100 ns. From Fig. 13a, it is clear that the RMSD value of HSA alone was stable from 0 to 40 ns, and it was increased from 40 to 60 ns and later became stable again from 60 to 100 ns. For HSA-T-res complex, the RMSD value steadily increases from 0 to 20 ns. From 40 to 100 ns, the system becomes stabilized, and no significant increase or decrease in the RMSD value is obtained, and the system reaches the equilibrium suggesting HSA and *trans*-resveratrol is steadily bound to HSA at subdomain IIA. During the simulated time of 100 ns, the RMSD value of HSA and *trans*-resveratrol complex was lesser than the RMSD value of HSA alone, this also suggests the stability of the HSA system after binding with *trans*-resveratrol molecule. The RMSD values of HSA obtained in the present study are in good agreement with the previously reported studies^55^.

Root mean square fluctuation (RMSF) values for HSA alone and HSA-*trans*-resveratrol complex were also evaluated to explore the protein flexibility^56^. Figure 13b shows the plot of RMSF values versus amino acid residues in HSA. It is inferred that the amino acid residues in subdomain IIA are more rigid than other regions due to the complex formation between HSA and *trans*-resveratrol. Owing to the presence of random coils at the end of helix, flexibility is observed at the end residues, as shown in Fig. 13b.

**Figure 14:**
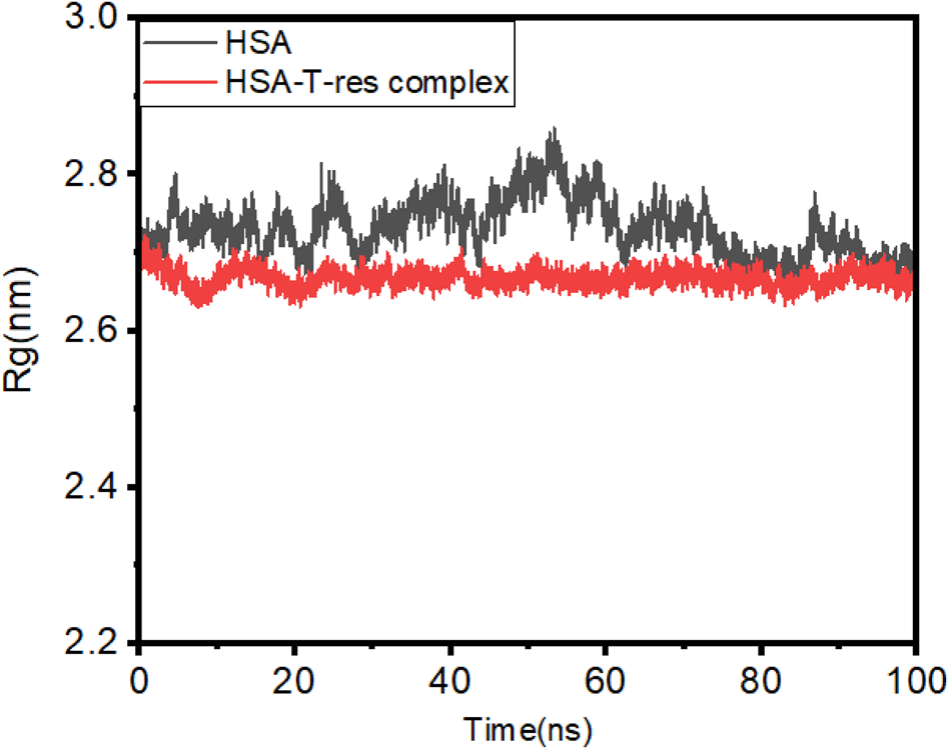
Radius of gyration plot from 0 to 100 ns time for HSA and HSA-T-res complex obtained from MD simulation studies.

**Figure 15:**
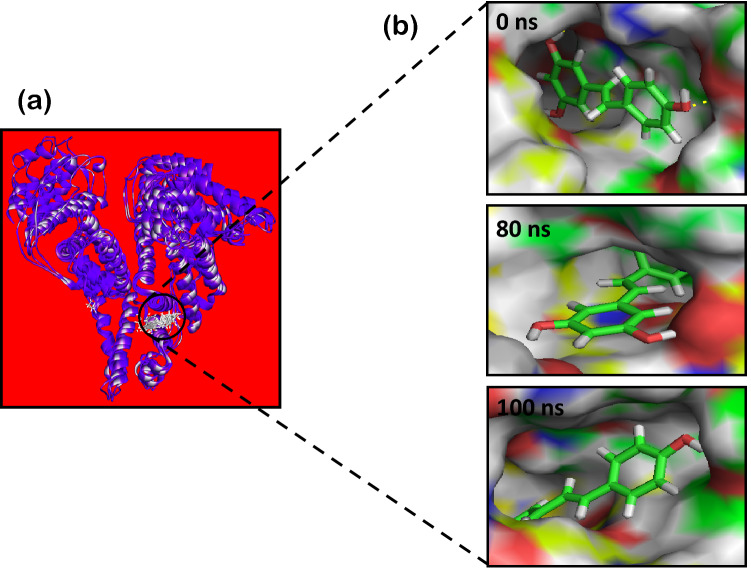
MD simulation results depicting **(a)** the cluster of *trans*-resveratrol molecule binding at subdomain IIA (Sudlow’s site 1) of HSA at 0, 80 and 100 ns, **(b)** surface view of the active site for the binding of *trans-*resveratrol to HSA obtained from MD simulation studies at 0, 80 and 100 ns simulation time.

Radius of gyration (R_g_) value for HSA alone and HSA-*trans*-resveratrol complex was calculated to investigate the compactness of the protein and structural dynamics of the system over the simulated period of 100 ns. From Fig. 14, it is evident that the R_g_ value of HSA alone initially fluctuates at around 2.80 nm and reaches a maximum fluctuation value of 2.85 nm at around 50 ns. After that, it decreases continuously up to 90 ns, and after that, it remains constant up to 100 ns. The R_g_ values of HSA obtained are in accordance with the previously reported studies^55,57^. On the other hand, the R_g_ value of HSA-*trans*-resveratrol complex initially decreases from 0 to 20 ns. Then it increases up to 30 ns, becomes stable, and reaches equilibrium from 30 to 100 ns. Figure 15 shows the dynamic nature of binding of *trans-*resveratrol to HSA at 0, 80, and 100 ns time at subdomain IIA. The findings of MD simulation studies corroborate the findings of our site markers displacement assays that also suggest subdomain IIA as the binding site of *trans*-resveratrol.

## Conclusion

The study describes the interacting potential of polyphenol, *trans*-resveratrol with HSA explored by UV-absorption spectroscopy, fluorescence spectroscopy, and molecular docking. The study also investigated the dislodging potential of *trans*-resveratrol competing for the binding site on HSA by displacing AFB_1_ bound to HSA using fluorescence spectroscopic tools. The fluorescence quenching study was used to calculate the Stern–Volmer quenching constant (K_SV_), binding constant (K_b_), and various thermodynamic parameters (ΔG, ΔH, and ΔS). The K_SV_ and K_b_ values obtained suggested high fluorescence quenching potential and strong binding constant, respectively, for *trans*-resveratrol and HSA. Circular dichroism studies confirmed an increase in the alpha-helix content in HSA in the presence of *trans*-resveratrol. MD simulation studies confirmed the dynamic nature of *trans*-resveratrol binding at the subdomain IIA of HSA over the simulated time of 0 to 100 ns. In silico amino acid substitution studies using Trp-214 mutated HSA provided the role of Trp-214 residue in the binding of *trans*-resveratrol to HSA.The thermal stability or melting temperature (Tm) of the HSA also increased in the presence of *trans*-resveratrol. The binding constant of AFB_1_ for serum albumin was lower than *trans*-resveratrol, signifying the displacing potential of the polyphenol for competing AFB_1_ acquiring the same binding site i.e. subdomain IIA or Sudlow’s site 1. This is the first investigation on the use of *trans*-resveratrol to remove AFB_1_ from serum albumin and thereby to decrease the bound form of AFB_1_. This is a spectroscopy-based study; however, an in vivo study is required to elucidate the effect of *trans-*resveratrol and AFB_1_ competition in animal models. The findings of this study will aid in the understanding of the pharmacokinetics and pharmacodynamics of phytochemical efficacy to compete with bound toxin and quick biotransformation leading to mycotoxin clearance from the body. The study will help explore more phytochemicals and their use in the unloading of the toxic substances from serum albumin, thus reducing the risk of pathological conditions in the human and animal body.

## Material and methods

### Materials

Aflatoxin B_1_, *trans*-resveratrol, human serum albumin, warfarin, and ibuprofen were procured from Sigma Aldrich (USA). The reagents used for making buffer solutions like sodium phosphate monobasic and sodium phosphate dibasic were obtained from SRL, India. All the chemicals were high purity grade and used as such without any further purification.

### Methods

#### Sample preparation

The stock solution of AFB_1_ of strength (1 mM) was constructed in HPLC grade methanol and later was diluted with 20 mM sodium phosphate buffer of pH 7.4 to maintain the working concentration. The stock solution of HSA (200 µM) was prepared in 20 mM sodium phosphate buffer, pH 7.4, and later it was diluted with the same buffer to make the working concentration. The stock solution of *trans*-resveratrol (1 mM) was prepared in HPLC grade methanol and distilled water in the ratio 1:1 (v/v) and later diluted with sodium phosphate buffer pH 7.4 to make the desired working concentrations.

#### UV-absorption studies

The UV-absorption studies were performed using Shimadzu UV-1900 spectrophotometer using quartz cuvettes of 1 cm. Sodium phosphate buffer of pH 7.4 and 20 mM strength was used for baseline correction and as a reference solution. The UV–absorption spectra of HSA, with the increasing concentration of *trans*-resveratrol, were recorded in the wavelength range of 220–360 nm. The concentration of HSA was fixed to (5 µM), and *trans*-resveratrol concentration was increased from (0–14 µM).

#### Fluorescence spectroscopic studies

Shimadzu RF-6000 spectrofluorometer equipped with xenon flash lamp was used for fluorescence spectroscopic studies. Initially, the binding potentials of AFB_1_ and *trans*-resveratrol for HSA were evaluated by studying the fluorescence emission spectrum of HSA in the presence of AFB_1_. HSA was excited at 280 nm, and emissions were recorded against increasing concentration of AFB_1_ (0–14 µM) in the wavelength range of 300–400 nm. The bandwidth of the excitation and emission wavelength was fixed to 5 nm each. The fluorescence intensity was corrected using Eq. (1) to check the inner filter effect

1 $${\text{Fcorr }} = {\text{ Fobs }} \times {\text{ e}}^{{\left( {{\text{Aex}} + {\text{Aem}}} \right)/{2}}}$$

In Eq. (1), Fcorr is the corrected fluorescence intensity; Fobs is the observed fluorescence intensity; Aex and Aem are the absorbances of ligand molecule at excitation and emission wavelength of HSA.

Binding parameters like Stern–Volmer quenching constant (K_SV_), binding constant (K_b_), and bimolecular quenching constant (K_q_) were calculated using the following Eq. (2) and (3).

2 $$\frac{\mathrm{F}_{0}}{\mathrm{F}}= 1+{\text{ K}}_{{{\text{sv}}}}[\mathrm{Q}] = {\text{K}}_{{\text{q}}} \tau_{0} (\mathrm{Q}) +1$$

3 $${\text{K}}_{{\text{q}}} = {\text{ K}}_{{{\text{sv}}}} / \, \tau_{0}$$

where F_0_ is the fluorescence intensity of HSA alone; F is the fluorescence intensity of HSA in the presence of ligand; Q is the concentration of the ligand; K_q_ is the bimolecular quenching constant, and τ_0_ is the average integral lifetime of the tryptophan residue in HSA (~ 10^–9^ s).The binding constant (K_b_) for the interaction of *trans*-resveratrol with HSA was calculated using Eq. (4)

4 $${\text{Log}} \frac{{{\text{F}}_{0} - {\text{F}}}}{{\text{F}}} = {\text{log K}}_{{\text{b}}} + {\text{ n log }}\left( {\text{Q}} \right)$$

5 $$\Delta {\text{G }} = \, - {\text{RTlnK}}_{{\text{b}}}$$

6 $${\text{ln K}}_{{\text{b}}} = \frac{\Delta S}{R} - \frac{\Delta H}{{RT}}$$

The calculations of thermodynamic parameters were based on Eqs. (5) and (6). In Eq. (5), ΔG is the Gibbs free energy, R is the universal gas constant (1.987 cal mol^−1^ K^−1^), T denotes temperature in kelvin, ΔS is the entropy change, and ΔH represents enthalpy change of the HSA and *trans*-resveratrol system.

#### Investigation of the binding site of *trans*-resveratrol on HSA using site markers

Most of the small molecule binds to HSA at Sudlow’s site 1 (subdomain IIA) or Sudlow’s site 2 (subdomain IIIA). To confirm the binding location of trans-resveratrol on HSA, a competitive site markers displacement assay was followed using warfarin and ibuprofen. Initially, 5 micro molar concentration of HSA was saturated with an excess of *trans*-resveratrol (14 µM) followed by titrations with increasing concentration of warfarin and ibuprofen (0–80 µM). The HSA and *trans*-resveratrol complex was excited at 280 nm, and the emission was recorded in the wavelength range of 300–400 nm. The percentage of *trans*-resveratrol displaced by the site markers is calculated according to Eq. (7) as follows

7 $${\text{Probe displacement }}\left( {\text{\% }} \right){ } = \frac{{{\text{F}}_{{2}} }}{{{\text{F}}_{{1}} }} \times 100\%$$

where F_1_ and F_2_ are the fluorescence intensities of HSA bound *trans*-resveratrol in the absence and presence of warfarin and ibuprofen site markers. The fluorescence spectrum was recorded using Shimadzu RF-6000 spectrofluorometer with fixed emission and excitation bandwidth of 5 nm each.

#### Circular dichroism

Jasco J-1500 spectropolarimeter was employed to investigate the secondary structure alterations in HSA in the presence of trans-resveratrol. The spectropolarimeter was equipped with a temperature control Peltier system. Quartz cuvette of path length 0.1 cm was used for taking the reading of the sample and for correcting the baseline. Far-UV CD spectra of HSA (5 µM) in the presence of *trans*-resveratrol (10 and 20 µM) were taken in the wavelength range of 190–250 nm with a data pitch of 1 nm. The scanning speed was fixed to 200 nm/min, and the bandwidth of 1 nm was set. Each spectrum was an average value of 3 spectra. MRE _208_ value and percentage alpha helix was calculated using Eqs. (8) and (9), respectively. The thermal melting profile of HSA in the presence of *trans*-resveratrol was studied by measuring the CD (mdeg) values at 222 nm at a temperature range of 20–90 °C, to investigate the melting temperature (T_m_) of HSA.

8 $${\text{MRE}}_{{208}} = \frac{{{\text{Observed}}\;{\text{CD}}\left( {{\text{mdeg}}} \right){\text{at}}\;208\;{\text{nm}}}}{{\left[ {C_{p} nl \times 10} \right]}}$$

9 $$\mathrm{Percentage } \, \alpha \, \mathrm{helix}= \frac{-{\mathrm{MRE}}_{208}-4000}{33000-4000}\times 100$$

In Eq. (8), C_p_ represents the concentration of the HSA, n is the number of amino acid residues in HSA, and l is the path length of the quartz cuvette in cm. in Eq. (9), MRE_208_ is the mean residual ellipticity value at 208 nm.

#### Competitive displacement of HSA bound AFB_1_ by trans-resveratrol

In the first set of experiments, the dislodging potential of *trans*-resveratrol was investigated to displace AFB_1_ from HSA bound to AFB_1_, employing the fluorescence spectroscopic tool. Five micromolar concentration of HSA was added to 14 µM AFB_1_ and subsequently titrated with the increasing concentration of *trans*-resveratrol (0–20 µM). The HSA-AFB_1_ complex was excited at 280 nm, and emission was recorded between 300 and 400 nm.

In the second set of experiments, the dislodging ability of AFB_1_ was explored by probing AFB_1_ ability to displace *trans*-resveratrol from HSA bound *trans*-resveratrol. The percentage displacement of the AFB or *trans*-resveratrol was studied by plotting F_2_/F_1_ × 100 versus the concentration of *trans*-resveratrol or AFB_1_.The experiment was performed using Shimadzu RF-6000 spectrofluorometer having xenon flash lamp, with excitation and emission bandwidth of 5 nm each. Sodium phosphate buffer of strength 20 mM and pH 7.4 was used to construct the desired concentration of the sample.

#### Molecular docking and in silico amino acid substitution studies

Autodock 4.2 tools were employed for performing molecular docking to explore the binding sites of *trans*-resveratrol on HSA. The chemical structure of *trans*-resveratrol was obtained from Pubchem (CID: 445154). The crystal structure of HSA was obtained from the RCSB protein data bank (PDB ID: 1AO6). The energy optimization of ligand was performed using Avogadro software, whereas Swiss PDB viewer was used for energy minimization of the protein molecule. PDBQT files were created for both ligand and protein after adding polar hydrogen and removing water molecules. Grid files were created with the grid dimension of 68 × 68 × 44 in xyz axis with grid point spacing of 0.375 Å, and all other parameters were used as default set values. Total 100 GA runs were processed for docking analysis, and Lamarckian genetic algorithm 4.2 with a maximum of 2,500,000 energy evaluations were used for docking calculations. In silico amino acid substitution studies were performed by substituting the desired amino acid in place of Trp-214 followed by saving the mutated HSA molecule in .pdb format. Molecular docking of the mutated HSA with *trans*-resveratrol was performed using the above-mentioned protocol for native HSA. The final docked complex was visualized using Discovery studio visualizer and PyMOL.

#### Molecular dynamic simulation studies

The MD simulation studies were carried out to explore the binding mode of *trans*-resveratrol to HSA. MD simulations were performed by Desmond v4.1 applied in Schrodinger-Maestro v11. The side-chain bumps and steric clashes were fixed. The PDB structure of HSA (1AO6) and *trans*-resveratrol (CID: 445154) were optimized for GROMOS96 54a7 force field. These prepared structures were then optimized by GROMOS96 54a7 force field^58^. Simple point charge water model was employed for adding the solvent molecules in the dodecahedron box with 1 Å distance from the protein surface, followed by the addition of four Na^+^ ions for the system neutralization. MD runs were set for 100 ns in three replicas with a time steps of 2 femtoseconds. The root mean square deviation (RMSD), root mean square fluctuation (RMSF) and radius of gyration (Rg) values were calculated post completion of the MD simulation runs.

#### Statistical analysis

Standard deviations (mean ± SD) were calculated wherever required in the experiment. One Way ANOVA analysis was conducted and the data with the p value < 0.05 were considered statistically significant. The statistical analysis was performed using OriginPro 2021 software.

## References

[1] Morabito, G., Miglio, C., Peluso, I. & Serafini, M. Fruit polyphenols and postprandial inflammatory stress. in *Polyphenols in Human Health and Disease*. Vol. **2**. 1107–1126. (Academic Press, 2014).

[2] Bertelli A (1999). Resveratrol, a natural stilbene in grapes and wine, enhances intraphagocytosis in human promonocytes: A co-factor in antiinflammatory and anticancer chemopreventive activity. Int. J. Tissue React..

[3] Weiskirchen S, Weiskirchen R (2016). Resveratrol: How much wine do you have to drink to stay healthy?. Adv. Nutr..

[4] Chang X, Heene E, Qiao F, Nick P (2011). The phytoalexin resveratrol regulates the initiation of hypersensitive cell death in Vitis cell. PLoS ONE.

[5] Salehi B (2018). Resveratrol: A double-edged sword in health benefits. Biomedicines.

[6] Xia N, Daiber A, Förstermann U, Li H (2017). Antioxidant effects of resveratrol in the cardiovascular system. Br. J. Pharmacol..

[7] de Coutinho DS, Pacheco MT, Frozza RL, Bernardi A (2018). Anti-inflammatory effects of resveratrol: Mechanistic insights. Int. J. Mol. Sci..

[8] Rege SD, Geetha T, Griffin GD, Broderick TL, Babu JR (2014). Neuroprotective effects of resveratrol in Alzheimer disease pathology. Front. Aging Neurosci..

[9] Vestergaard M, Ingmer H (2019). Antibacterial and antifungal properties of resveratrol. Int. J. Antimicrob. Agents.

[10] Sridhar M, Suganthi RU, Thammiaha V (2015). Effect of dietary resveratrol in ameliorating aflatoxin B1-induced changes in broiler birds. J. Anim. Physiol. Anim. Nutr. (Berl).

[11] Reiter E, Zentek J, Razzazi E (2009). Review on sample preparation strategies and methods used for the analysis of aflatoxins in food and feed. Mol. Nutr. Food Res..

[12] Gizachew D, Chang C, Szonyi B, De La Torre S, Ting W (2019). Aflatoxin B1 (AFB1) production by *Aspergillus flavus* and *Aspergillus parasiticus* on ground Nyjer seeds: The effect of water activity and temperature. Int. J. Food Microbiol..

[13] Kumar P, Mahato DK, Kamle M, Mohanta TK, Kang SG (2016). Aflatoxins: A global concern for food safety, human health and their management. Front. Microbiol..

[14] Williams J (2004). Human aflatoxicosis in developing countries: A review of toxicology, exposure, potential health consequences, and interventions. Am. J. Clin. Nutr..

[15] Paul S, Ghanti R, Sardar PS, Majhi A (2019). Synthesis of a novel coumarin derivative and its binding interaction with serum albumins. Chem. Heterocycl. Compd..

[16] Aamir Qureshi M, Javed S (2020). Structural dynamics studies on the binding of aflatoxin B1 to chicken egg albumin using spectroscopic techniques and molecular docking. J. Biomol. Struct. Dyn..

[17] Poór M (2017). Investigation of non-covalent interactions of aflatoxins (B1, B2, G1, G2, and M1) with serum albumin. Toxins Basel.

[18] Qureshi MA, Javed S (2021). Aflatoxin B1 induced structural and conformational changes in bovine serum albumin: A multispectroscopic and circular dichroism-based study. ACS Omega.

[19] Gadallah MI, Ali HRH, Askal HF, Saleh GA (2020). Towards understanding of the interaction of certain carbapenems with protein via combined experimental and theoretical approach. Spectrochim. Acta Part A Mol. Biomol. Spectrosc..

[20] Rasoulzadeh F, Asgari D, Naseri A, Rashidi MR (2010). Spectroscopic studies on the interaction between erlotinib hydrochloride and bovine serum albumin. DARU J. Pharm. Sci..

[21] Phopin K, Ruankham W, Prachayasittikul S, Prachayasittikul V, Tantimongcolwat T (2020). Insight into the molecular interaction of cloxyquin (5-chloro-8-hydroxyquinoline) with bovine serum albumin: Biophysical analysis and computational simulation. Int. J. Mol. Sci..

[22] Siddiqui S (2021). Biophysical insight into the binding mechanism of doxofylline to bovine serum albumin: An in vitro and in silico approach. Spectrochim. Acta Part A Mol. Biomol. Spectrosc..

[23] Singh I, Luxami V, Paul K (2020). Spectroscopy and molecular docking approach for investigation on the binding of nocodazole to human serum albumin. Spectrochim. Acta Part A Mol. Biomol. Spectrosc..

[24] Ai Zhi W, Chao Zhan L, Ya Jing Z, Jia Lin Z, Chen Chen Z (2013). Investigation of the interaction between two phenylethanoid glycosides and bovine serum albumin by spectroscopic methods. J. Pharm. Anal..

[25] Amir M, Qureshi M, Javed S (2021). Biomolecular interactions and binding dynamics of tyrosine kinase inhibitor erdafitinib, with human serum albumin. J. Biomol. Struct. Dyn..

[26] Kandagal PB (2006). Study of the interaction of an anticancer drug with human and bovine serum albumin: Spectroscopic approach. J. Pharm. Biomed. Anal..

[27] Zhang J, Gao X, Huang J, Wang H (2020). Probing the interaction between human serum albumin and 9-hydroxyphenanthrene: A spectroscopic and molecular docking study. ACS Omega.

[28] Goszczyński TM, Fink K, Kowalski K, Leśnikowski ZJ, Boratyński J (2017). Interactions of boron clusters and their derivatives with serum albumin. Sci. Rep..

[29] Wang BL, Pan DQ, Zhou KL, Lou YY, Shi JH (2019). Multi-spectroscopic approaches and molecular simulation research of the intermolecular interaction between the angiotensin-converting enzyme inhibitor (ACE inhibitor) benazepril and bovine serum albumin (BSA). Spectrochim. Acta Part A Mol. Biomol. Spectrosc..

[30] Wang Q (2016). Binding interaction of atorvastatin with bovine serum albumin: Spectroscopic methods and molecular docking. Spectrochim. Acta Part A Mol. Biomol. Spectrosc..

[31] Zhang Y-F, Zhou K-L, Lou Y-Y, Pan D, Shi J-H (2016). Investigation of the binding interaction between estazolam and bovine serum albumin: Multi-spectroscopic methods and molecular docking technique. J. Biomol. Struct. Dyn..

[32] Abdelaziz MA, Shaldam M, El-Domany RA, Belal F (2022). Multi-Spectroscopic, thermodynamic and molecular dynamic simulation studies for investigation of interaction of dapagliflozin with bovine serum albumin. Spectrochim. Acta Part A Mol. Biomol. Spectrosc..

[33] Tan H (2019). Fluorescence spectroscopic investigation of competitive interactions between quercetin and aflatoxin B1 for binding to human serum albumin. Toxins (Basel).

[34] Ross PD, Subramanian S (1981). Thermodynamics of protein association reactions: Forces contributing to stability. Biochemistry.

[35] Su X, Wang L, Xu Y, Dong L, Lu H (2021). Study on the binding mechanism of thiamethoxam with three model proteins:Spectroscopic studies and theoretical simulations. Ecotoxicol. Environ. Saf..

[36] Sudlow G, Birkett DJ, Wade DN (1975). The characterization of two specific drug binding sites on human serum albumin. Mol. Pharmacol..

[37] Gholivand MB, Jalalvand AR, Goicoechea HC, Omidi M (2013). Investigation of interaction of nuclear fast red with human serum albumin by experimental and computational approaches. Spectrochim. Acta Part A Mol. Biomol. Spectrosc..

[38] Wu D, Wang J, Liu D, Zhang Y, Hu X (2019). Computational and spectroscopic analysis of interaction between food colorant citrus red 2 and human serum albumin. Sci. Rep..

[39] Liu T (2020). Investigation of binary and ternary systems of human serum albumin with oxyresveratrol/piceatannol and/or mitoxantrone by multipectroscopy, molecular docking and cytotoxicity evaluation. J. Mol. Liq..

[40] Greenfield NJ (2006). Using circular dichroism spectra to estimate protein secondary structure. Nat. Protoc..

[41] Wallace BA (2000). Conformational changes by synchrotron radiation circular dichroism spectroscopy. Nat. Struct. Biol..

[42] Louis-Jeune C, Andrade-Navarro MA, Perez-Iratxeta C (2012). Prediction of protein secondary structure from circular dichroism using theoretically derived spectra. Proteins Struct. Funct. Bioinform..

[43] Martin S, Schilstra M (2008). Circular dichroism and its application to the study of biomolecules. Methods Cell Biol..

[44] Miles AJ, Wallace BA (2016). Circular dichroism spectroscopy of membrane proteins. Chem. Soc. Rev..

[45] Liao X (2021). Investigation on the binding of cyanobacterial metabolite calothrixin A with human serum albumin for evaluating its potential toxicology. Food Chem. Toxicol..

[46] Jiao Q (2019). Study on the interactions between caffeoylquinic acids with bovine serum albumin: Spectroscopy, antioxidant activity, LC-MSn, and molecular docking approach. Front. Chem..

[47] Wu LL, Gao HW, Gao NY, Chen FF, Chen L (2009). Interaction of perfluorooctanoic acid with human serum albumin. BMC Struct. Biol..

[48] Das S (2017). Molecular binding of toxic phenothiazinium derivatives, azures to bovine serum albumin: A comparative spectroscopic, calorimetric, and in silico study. J. Mol. Recognit..

[49] Dill KA (2002). Dominant forces in protein folding. Biochemistry.

[50] Novokhatny V, Ingham K (1997). Thermodynamics of maltose binding protein unfolding. Protein Sci..

[51] Miotto M (2019). Insights on protein thermal stability: A graph representation of molecular interactions. Bioinformatics.

[52] Lohner K, Sen A, Prankerd R, Esser A, Perrin J (1994). Effects of drug-binding on the thermal denaturation of human serum albumin. J. Pharm. Biomed. Anal..

[53] Chatterjee T, Pal A, Dey S, Chatterjee BK, Chakrabarti P (2012). Interaction of virstatin with human serum albumin: Spectroscopic analysis and molecular modeling. PLoS ONE.

[54] Meng X-Y, Zhang H-X, Mezei M, Cui M (2011). Molecular docking: A powerful approach for structure-based drug discovery. Curr. Comput. Aided. Drug Des..

[55] Guizado TRC, Louro SRW, Anteneodo C (2012). Dynamics of heme complexed with human serum albumin: A theoretical approach. Eur. Biophys. J..

[56] Niu X, Gao X, Wang H, Wang X, Wang S (2013). Insight into the dynamic interaction between different flavonoids and bovine serum albumin using molecular dynamics simulations and free energy calculations. J. Mol. Model..

[57] Soltanabadi O, Atri MS, Bagheri M (2018). Spectroscopic analysis, docking and molecular dynamics simulation of the interaction of cinnamaldehyde with human serum albumin. J. Incl. Phenom. Macrocycl. Chem..

[58] Schmid N (2011). Definition and testing of the GROMOS force-field versions 54A7 and 54B7. Eur. Biophys. J..

